# Return to work factors and vocational rehabilitation interventions for long-term, partially disabled workers: a modified Delphi study among vocational rehabilitation professionals

**DOI:** 10.1186/s12889-022-13295-6

**Published:** 2022-05-02

**Authors:** Christa J. C. de Geus, Maaike A. Huysmans, H. Jolanda van Rijssen, Johannes R. Anema

**Affiliations:** 1 Department of Public and Occupational Health, Amsterdam Public Health Research Institute, Amsterdam UMC, Vrije Universiteit Amsterdam, Van der Boechorststraat 7, NL 1081 BT Amsterdam, the Netherlands; 2 Research Centre for Insurance Medicine, AMC-UMCG-VUmc-UWV, Amsterdam, the Netherlands; 3 Dutch Institute of Employee Benefit Schemes (UWV), Amsterdam, the Netherlands

**Keywords:** Decision aid, Disability pension, Labour experts, Insurance physicians, Long-term sick leave, Return to work

## Abstract

**Background:**

Long-term disability has a great impact on both society and workers with disabilities. Little is known about the barriers which prohibit workers with long-term disabilities from returning to work and which interventions are best suited to counteract these barriers. The main purpose of this study was to obtain consensus among professionals on important return to work (RTW) factors and effective vocational rehabilitation (VR) interventions for long-term (> 2 years), partially disabled workers. Our three research questions were: (1) which factors are associated with RTW for long-term disabled workers?; (2) which factors associated with RTW can be targeted by VR interventions?; and (3) which VR interventions are the most effective to target these factors?

**Methods:**

A modified Delphi Study was conducted using a panel of 22 labour experts, caseworkers, and insurance physicians. The study consisted of several rounds of questionnaires and one online meeting.

**Results:**

The multidisciplinary panel reached consensus that 58 out of 67 factors were important for RTW and that 35 of these factors could be targeted using VR interventions. In five rounds, the expert panel reached consensus that 11 out of 22 VR interventions were effective for at least one of the eight most important RTW factors.

**Conclusions:**

Consensus was reached among the expert panel that many factors that are important for the RTW of short-term disabled workers are also important for the RTW of long-term partially disabled workers and that a substantial number of these factors could effectively be targeted using VR interventions. The results of this study will be used to develop a decision aid that supports vocational rehabilitation professionals in profiling clients and in choosing suitable VR interventions.

**Supplementary Information:**

The online version contains supplementary material available at 10.1186/s12889-022-13295-6.

## Background

Long-term work disability has many negative consequences for the lives of disabled workers. Not being able to return to work leads to economic and social deprivation [1] and reduced psychological well-being [2]. Workers who take long leaves of absence from work likewise have a lower chance of returning [1]. In the long run, only 20% of people who receive disability benefits for longer than six months will return to work (RTW) [1]. Such long-term absences not only influence the lives of the disabled workers, but also result in increased financial burden at a societal level. In 2019, 12.8 billion euros was spent on disability benefits in the Netherlands alone [3].

Thus, both individuals and society would benefit if partially disabled workers were able to return to work sooner. However, these workers often cannot return to work on their own. After two years of sick leave, workers in the Netherlands can apply for a disability benefit at the Dutch Social Security Institute (SSI). Workers who receive this benefit and who are still capable of earning part of their income receive help from the SSI with returning to work. At the SSI, labour experts and caseworkers try to support disabled workers in their RTW by referring them to vocational rehabilitation (VR) interventions [4]. To see which VR interventions are the best fit for a given disabled worker, labour experts and caseworkers consider the factors prohibiting the worker from returning to work and the factors which may promote their RTW. Currently, the assessments made by labour experts and caseworkers are not evidence-based, but rather practice-based due to insufficient scientific knowledge regarding factors that are barriers for RTW and VR interventions that are effective. This means that whether factors are seen as barriers, how these barriers are assessed, and which VR interventions are offered to a disabled worker, will vary between professionals (i.e., individual labour experts or caseworkers). Part of this variation cannot be explained and is unneeded and therefore undesirable variation, leading to offering not well-founded VR interventions.

To increase evidence-based knowledge on this topic and increase the effectivity of care delivered by labour experts, the development of evidence-based assessment tools and evidence-based VR interventions is needed. Whereas a number of studies have investigated which factors play a role in the RTW of workers after short-term sick leave (for example [5, 6]), only one study could be found which examined the factors of importance for long-term (> 2 years) disabled workers [7]. Scientific evidence is also limited regarding the VR interventions effective in facilitating RTW for long-term disabled workers. Several recent systematic reviews describe a variety of interventions for disabled workers [8, 9], but many of these interventions are not effective or have only proved to be effective for a specific patient group [8]. Interventions that are effective across patient groups are still missing.

There is a lack of scientific evidence on RTW for long-term disabled workers. A first step in developing an evidence base is collecting and combining practice-based knowledge from professionals with considerable expertise in this field.

This study aims to retrieve practice-based evidence, by reaching consensus among a multidisciplinary panel of labour experts, caseworkers, and insurance physicians on which factors influence RTW of long-term (> 2 years) partially disabled workers and which VR interventions are effective in targeting these factors. We examined which factors and VR interventions are important for the RTW of long-term (> 2 years), partially disabled workers. The three research questions of this study were: (1) which factors are associated with the RTW of long-term disabled workers?; (2) which factors associated with RTW can be targeted with VR interventions?; and (3) which VR interventions are most effective for targeting these factors?

## Methods

**Table:** 

Context:
In the Netherlands, people who have been on sick leave for two years can apply for a work disability pension at the Dutch Social Security Institute (SSI) based on the Act on Work and Income According to Work Capacity (WIA). People that receive a WIA-work disability pension and who still have (partial) work capacity are expected to earn a part of their income. To achieve this goal, these persons receive support from the SSI on returning to work. This support is offered by labour experts. In a face-to-face interview, the labour expert assesses the barriers that need to be addressed and the VR interventions that facilitate RTW for a specific person. Based on this interview, a rehabilitation plan is developed and the person with partial work capacity is referred to a matching rehabilitation provider to receive a VR intervention. The labour expert monitors the progress of RTW.

### Study design

In this study, we used a stepwise modified Delphi technique [10] by combining the Delphi technique [11, 12] and the nominal group technique [13] to obtain multidisciplinary consensus from our expert panel. The panel consisted of labour experts, caseworkers, and insurance physicians. The Delphi consisted of several rounds using online questionnaires and an online meeting which employed the nominal group technique. The nominal group technique was used to structure the meeting to explore differences between individual labour experts and caseworkers in their opinion about RTW barriers and interventions and to reach consensus among the participants by group discussions [13]. Earlier studies have successfully used the combination of the Delphi technique and nominal group technique [14, 15].

At the start of the Delphi study, we formed a list of potential RTW factors and VR interventions by searching through scientific studies and semi-scientific (grey) literature on related populations and by speaking with professionals. We retrieved 58 RTW factors from semi-scientific studies [16, 17], scientific reviews [18–21], and conversations with professionals working at the SSI. VR interventions were retrieved through an analysis of which interventions are currently offered to long-term disabled workers in the Netherlands. These were supplemented with VR interventions found in scientific [22–28] and semi-scientific [29] literature. To search for VR interventions within academic literature, we used the following definition: "a multi-professional evidence-based approach that is provided in different settings, services, and activities to working age individuals with health-related impairments, limitations, or restrictions with work functioning, and whose primary aim is to optimize work participation" [30].

### Study population

Our panel of experts included people who had been working for at least one year as a labour expert, a caseworker, or an insurance physician with the RTW of long-term disabled workers at the Dutch SSI.

We recruited the panel of labour experts and caseworkers via a recruitment message distributed in the SSI’s internal newsletter. Out of the responses to the recruitment message, we selected labour experts and caseworkers who were representative of different regions, ages, genders, and years of experience. In addition, we recruited insurance physicians with experience in the rehabilitation of long-term sick people via an email message.

### Data collection and analysis

The Delphi study consisted of three sub-questions which experts answered over the course of five Delphi questionnaire rounds. An overview of these rounds and consensus rules can be found in Table 1.

**Table 1:** Overview of questions asked in each round

	**Number of experts**	**1. Which factors are associated with the RTW of long-term disabled workers?**	**2. Which of these identified RTW factors can be targeted using VR interventions?**	**3. What are the most effective VR interventions to target these factors?**
Round 1	21	Scoring 58 RTW factors retrieved from literature and practice on their association with RTW. Answer options were (1) totally disagree – (5) totally agree - ≥ 80% (4) agree or (5) totally agree accepted - ≥ 70%—80% (4) agree or (5) totally agree consensus not reached and presented again in the next round - < 70% (4) agree or (5) totally agree left out of remaining rounds Option to add new relevant RTW factors	Scoring 58 RTW factors on whether they can be influenced by VR interventions. Answer options were (1) yes or (2) no - ≥ 80% (1) yes accepted - ≥ 70%—80% (1) yes consensus not reached and presented again in the next round - < 70% (1) yes left out of remaining rounds	N/A
Round 2	20	Scoring RTW factors for which consensus was not reached in round 1 - ≥ 80% (4) agree or (5) totally agree accepted - < 80% (4) agree or (5) totally agree left out of remaining rounds Scoring the newly added RTW factors in round 1 on their association with RTW - Same answering categories and consensus rules as in round 1	Scoring RTW factors for which consensus was not yet reached - ≥ 80% (1) yes accepted - < 80% (1) yes left out of remaining rounds Scoring newly added RTW factors - Same answering categories and consensus rules as in round 1	N/A
Round 3	20	Scoring newly added factors for which consensus was not reached in round 2 - ≥ 80% (4) agree or (5) totally agree accepted - < 80% (4) agree or (5) totally agree left out of remaining rounds	Scoring newly added factors for which consensus was not yet reached - ≥ 80% (1) yes accepted - < 80% (1) yes left out of remaining rounds	N/A
Round 4	19	N/A	N/A	Scoring VR interventions for factors for which consensus was reached for questions 1 and 2. Answer options were (1) not effective, (2) somewhat effective, and (3) very effective - ≥ 70% (3) very effective accepted - ≥ 50% and < 70% (3) very effective consensus not reached and presented again in the next round - < 50% very effective left out of remaining round
Round 5	20	N/A	N/A	Due to time constraints, we limited the number of factors to be discussed. Therefore, for the fifth round we selected RTW factors for which ≥ 70% of the experts (5) totally agreed that the factor was important for RTW, and for which there was consensus that this factor could be influenced with a VR intervention (round 2). This resulted in a total of eight factors. For these factors VR interventions were scored for which consensus was not yet reached in round 4. Answer options were (1) not effective or (2) somewhat or very effective - ≥ 70% (2) somewhat or very effective accepted - < 70% intervention is seen as ineffective in targeting a factor

#### Sub-questions

##### Factors associated with the RTW of long-term disabled workers (Rounds 1, 2, 3)

In the first question, experts were asked to indicate to what extent they agreed with the statement “This factor influences the RTW of long-term disabled workers”. Experts could rate the extent of their agreement on a 5-point Likert scale. The response options were: (1) totally disagree, (2) disagree, (3) neutral, (4) agree, and (5) totally agree. Consensus was reached if ≥ 80% of the experts scored (4) agree or (5) totally agree for a particular factor. These factors were accepted without further discussion. If the consensus percentage was between ≥ 70% and < 80%, the RTW factor had to be scored again by the experts in the next questionnaire round. If the consensus percentage was lower than 70%, the factor was not considered to be important for the RTW of long-term disabled workers and was left out of the study. Experts also had the option of adding factors which they found to be important for the RTW of this group that were not on the list. These new factors were added to the second round, leaving out duplicates. In the second round, experts were once again asked to indicate to what extent they agreed with the newly added factors and the factors for which no consensus was reached in the previous round. Per factor, experts were provided with an overview of the number of participants that choose a specific response category for this question in the previous round and their own response.Afterward, the consensus percentage was calculated in the same way as in the first round. Factors for which the consensus percentage was between ≥ 70% and < 80% after the second round were scored again in the third round.

##### Factors that can be targeted by VR interventions (Rounds 1, 2, 3)

In the second question, experts were asked to indicate if they thought that a factor could be targeted with an intervention aimed at returning to work. The response options were: (1) yes or (2) no. Consensus was established if ≥ 80% of the experts scored (1) yes or (2) no. These factors were accepted without further discussion. Factors that had a consensus percentage < 70% were considered not to be targetable with VR interventions. As in the first question, if the consensus percentage was between ≥ 70% and < 80% the RTW factor was scored again by the experts in the next questionnaire. In the second and third rounds, experts were provided with an overview of the number of participants that choose a specific response category for this question in the previous round and their own response.In the third round, the newly added factors for which there was no agreement reached in the second round were once more scored by the experts.

##### Effective VR interventions to target RTW factors (Rounds 4 and 5)

Finally, the factors which had been determined by consensus to be (a) associated with the RTW of long-term disabled workers and (b) that could be targeted by using VR interventions were once more presented to the experts in a fourth round. Experts were asked to indicate to what extent 22 clusters of VR interventions would be effective in targeting each of the factors. These clusters of VR interventions were collected in (academic) literature and in practice. Experts received a short description of the VR interventions and could indicate their answer on a 3-point Likert scale. The response options were: (1) not effective, (2) somewhat effective, and (3) very effective. After this round, the consensus percentages were calculated for each intervention on each factor. VR interventions for which ≥ 70% of the experts scored (3) very effective were established to be targetable with RTW factors and were accepted without further discussion. VR interventions for which ≥ 50% and < 70% of the experts had scored the intervention to be (3) very effective were once more presented to the experts to be scored in the next round. VR interventions for which < 50% scored the intervention (3) very effective were excluded from subsequent Delphi rounds.

The fifth round consisted of an online meeting and online questionnaire. Due to a combination of the large number of VR interventions for the factors for which consensus was not reached in the fourth round, the large number of potential interventions, and time constraints, only a selection of factors and their VR interventions could be discussed and scored again. We selected the 8 factors with the highest consensus percentage for the statement: “this factor is associated with RTW of long-term disabled workers”, to reach consensus on the most effective VR interventions to target these factors for the VR interventions for which consensus was not yet reached.

The VR interventions for the other factors were not scored again in the fifth round. The results of the fourth round were thus the final results for the VR interventions of these RTW factors. RTW factors for which ≥ 70% of the experts scored (5) totally agree for the factor being important for RTW, and for which there was consensus that this factor could be influenced with a VR intervention (round 2) were taken to the fifth round. Participants received an overview of the percentage of participants that choose a certain response option in the previous round.

In the online meeting each factor was presented on the screen and with an online voting tool participants rated all VR interventions (for which no consensus had been reached in round four) on whether they were (1) not effective or (2) somewhat/very effective to target the factor. Then the results were discussed with all participants. The results of this discussion were incorporated into the final online questionnaire. Only half of the participants could attend this meeting. The other participants received the questions on paper.

In the final online questionnaire, all participants (including those not present during the online meeting) were asked to once again rate all VR interventions (for which no consensus had been reached in round four) on a dichotomous scale of whether or not they were effective to target each of the eight RTW factors. Consensus in this round was reached if ≥ 70% of the experts agreed that the intervention was (2) somewhat/very effective in targeting a particular factor. These VR interventions were accepted without further discussion.

## Results

### Expert panel

In total, 22 experts (ten labour experts, nine caseworkers, and three insurance physicians) agreed to participate in the Delphi study. Before the start of the first round, one participant withdrew from the study due to lack of time and a second participant withdrew from the study due to illness after the first round. One participant failed to fill out the questionnaire in the fourth round. See Table 1 for the number of experts per round.

### Delphi rounds

#### Which factors are associated with the RTW of long-term partially disabled workers? (Rounds 1, 2, 3)

The results of round 1 found 80% of the experts to agree that 44 out of the 58 factors were indeed associated with the RTW of long-term disabled workers. Seven factors received a consensus percentage between 70–80% in the first round, six of which reached consensus in the second round. For eight factors, less than 70% of the experts agreed that the factor was associated with the RTW of long-term disabled workers. These factors were left out the Delphi after the first two rounds.

In the first round, the experts added 50 suggestions for additional factors. Based on these suggestions and after removing any duplicates, nine new factors were formulated. In round two, consensus was reached that five out of these nine new factors were associated with the RTW of long-term disabled workers. The four additional factors had an agreement percentage of between 70–80% and were once again presented to experts in the third round. In round three, consensus was reached for another three factors. After three rounds, the experts agreed that 58 out of the 67 factors were associated with the RTW of long-term partially disabled workers. An overview of the factors that were found to be associated with the RTW of long-term partially disabled workers can be found in Table 2. An overview of the consensus percentage per round can be found in Supplementary Material 1.

**Table 2:** Overview of important RTW factors and effective VR interventions according to our expert panel

Factors associated with RTW (> 80%)	Factor that can be targeted using VR interventions (> 80%)	VR interventions that are effective in targeting a factor (> 70%)
Motivation to RTW [18]	yes	E (89%)^1^, G (95%)^2^, I (90%)^2^, R (90%)^2^, F (85%)^2^, K (85%)^2^, L (80%)^2^, H (75%)^2^
Illness perception^b^	yes	G(72%)^1^, H (95%)^2^, I (95%)^2^
Societal participation^b^	yes	F (95%)^1^, E (95%)^2^, R (95%)^2^, G (85%)^2^, H (70%)^2^
Importance of work [18]	yes	F (90%)^2^, G (90%)^2^, R (90%)^2^, K (85%)^2^, E (85%)^2^, L (70%)^2^
Family issues^b^	yes	D (74%)^1^, H (85%)^2^, G (80%)^2^, U (70%)^2^
Financial problems^b^ [17]	yes	D (95%)^1^
History of substance abuse^b^	yes	None
Recent life events^a^	yes	H (90%)^2^, D (85%)^2^, T (80%)^2^
Job application skills^b^	yes	N (89%)^1^
Coping^b^	yes	H (100%)^2^, G (90%)^2^, E (85%)^2^, I (85%)^2^, T (80%)^2^
Job self-efficacy [16]	yes	G (72%)^1^, I (72%)^1^, L (72%)^1^, R (72%)^1^
Fear avoidance behaviour [19]	yes	G (83%)^1^
Social network [16]	yes	F (84%)^1^
Willing to make concessions [16]	yes	None
Reintegration services already started in the past^a^	yes	None
Employee skills [17]	yes	J (83%)^1^
RTW self-efficacy [18]	yes	H (72%)^1^, L (72%)^1^, R (72%)^1^
Self-esteem [17]	yes	G (79%)^1^
RTW expectations [16, 18]	yes	None
Self-sufficiency^b^	yes	None
Quality of life [19]	yes	None
Unhealthy lifestyle [16]	yes	None
Caring for children^a^	yes	None
Experiences at old workplace^b^	yes	None
Unemployment[17]	yes	Not applicable
Perceived general health [16, 21]	yes	G (78%)^1^, I (78%)^1^, H (72%)^1^
Work-life balance^b^	yes	None
Sense of responsibility^b^	yes	None
Work ability [17, 18]	yes	None
Previously been in contact with the law	yes	None
Knowledge of the labour market^a^	yes	L (78%)^1^
Secondary gain of illness^a^	yes	E (79%)^1^
Job search behaviour^b^	yes	N (83%)^1^, L (78%)^1^
Job search intensity [16]	yes	L (72%)^1^, M (72%)^1^
Volunteer work [17]	yes	Not applicable
Understanding of the Dutch language^b^	no	
Language proficiency^b^	no	
Social support (outside of work) [18]	no	
Alcohol/Substance abuse [17]	no	
Secure housing^a^	no	
Compensation claim for personal injury^a^	no	
Diplomas^b^	no	
Main wage earner [17, 21]	no	
Pain [19–21]	no	
Treatment^b^	no	
Income [21]	no	
Willingness to learn^b^	no	
Disability rate^b^	no	
Social norms regarding RTW [16]	no	
Transportation^b^	no	
Objection or appeal to decision for disability pension^b^	no	
Work history^b^	no	
Level of education [18]	no	
(Re-)training^b^	no	
Health transition [19]	no	
(Informal) Care [17]	no	
Age [16, 18, 20, 21]	no	
Time since last working day^a^	no	

^a^Factor added based on suggestions by experts in round 1

^b^Factor was retrieved from conversations with professionals

^c^Factors were ranked based on 1) if they could be targeted with a VR intervention and 2) the percentage of consensus if a factor is associated with the RTW of a long-term disabled worker

^1^Consensus was reached in round 4 VR interventions for which consensus was reached in round 4 are listed before VR interventions for which consensus was reached in the fifth round

^2^Consensus was reached in round 5

(A) Informing the disabled worker about the disability benefit or the re-integration process; (B) Professional (multidisciplinary) consultation to optimize the service offered to the disabled worker; (C) Assessing vocational needs; (D) Referral to services offered by other organizations; (E) Increasing motivation; (F) Improving societal participation; (G) Improving self-image and self-knowledge; (H) Increasing psychological resilience; (I) Improving vitality and physical resilience; (J) Strengthening employee skills; (K) Identifying what the disabled worker wants to do in terms of work; (L) Identifying what the disabled worker can do in terms of work; (M) Helping to search for vacancies; (N) Improving skills and helping with applying for a job; (O) Mediating; (P) Workplace adjustments or support; (Q) Training; (R) Increasing work experience; (S) Providing facilities; (T) Cognitive behavioural therapy; (U) Multidisciplinary interventions; (V) Individual Placement and Support (IPS). A description of the VR interventions can be found in Supplement 3. The VR interventions were assigned a letter based on the order in which we presented them to the participants during the Delphi

#### Which of the identified RTW factors can be targeted using VR interventions? (Rounds 1, 2, 3)

In the first round, consensus was reached (> 80% of the experts) that 24 factors could be targeted using VR interventions. Seven factors scored between 70–80% and were scored again by experts in the second round. Consensus was then reached for six additional factors. In the second round, experts also scored the new nine factors which had been added and reached agreement for four out of the nine. (Round 3) A fifth new factor scored between 70–80% and was thus scored again in the third round (where agreement on this factor was reached). In total, experts agreed that 35 out of the 67 RTW factors that were found in the search or added by the experts in the first round could be targeted using VR interventions. Table 2 shows the factors which could be targeted using a VR intervention according to the consensus reached. An overview of the consensus percentages per round can be found in Supplementary Material 2.

#### What are the most effective VR interventions to target RTW factors? (Rounds 4 and 5)

After the first three rounds, there was consensus among the experts that 35 factors (a) could be targeted in order to increase the chances of RTW for long-term disabled workers and (b) could be influenced with a VR intervention. Thirty-three of these factors were presented to the experts in the fourth round. Two factors, ‘unemployment’ and ‘volunteer work’, were left out of because they overlapped with the clusters of VR interventions. The experts were asked to indicate which VR interventions were effective in targeting each factor. After the fourth round, 26 effective VR interventions were found.

In the fifth and final Delphi round, the experts were asked to once more give their opinion on the effectiveness of the types of VR interventions for the selection of eight factors. These factors were selected based on that consensus was reached that they were important for the RTW (≥ 70% (5) totally agreed that the factor was important for RTW), and for which there was consensus that this factor could be targeted by using a VR intervention. For these eight factors, only the VR interventions for which consensus had not been reached in the fourth round were selected for the fifth round. The fifth round started with an online meeting in which experts (*n* = 10) discussed their opinions on the topic. In the online questionnaire, experts (*n* = 20) reached consensus for 30 VR interventions for the eight factors. In total (for all factors and including the VR interventions for which consensus was reached in round 4), the experts found 56 VR interventions to be effective. The experts agreed that multiple types of VR interventions could be effective per factor. For example, for the factor ‘motivation’ consensus was reached that eight types of VR intervention could be effective. Likewise, certain VR interventions were found to be effective for multiple RTW factors. For some factors, none of the proposed VR interventions were found to be effective. An overview of the VR interventions that could target RTW factors (according to the consensus) can be found in Table 2.

## Discussion

### Main findings

The main aim of this Delphi study was to obtain multidisciplinary consensus among labour experts, caseworkers, and insurance physicians working at the Dutch SSI on which factors were relevant for the RTW of long-term (> 2 years) partially disabled workers and which VR interventions can be effective in improving RTW for this group. The factors were retrieved from literature reviews or practice. Among the experts, consensus was reached that 58 factors affect the RTW of long-term disabled workers. Furthermore, consensus was reached that 35 of these 58 factors could be targeted using a VR intervention. There was no consensus as to whether the other 23 factors could be influenced with a VR intervention because they were more or less fixed (for example, sex or age) or because a different types of intervention were needed (for example language proficiency). Finally, consensus was reached on what the most effective VR interventions were for the eight most important factors for RTW. For each of these factors, between one and eight interventions could be effective in increasing RTW according to the experts. Out of the 22 possible interventions, 11 interventions were effective for at least one of these eight factors.

### Comparison with other studies

#### Factors

The 58 factors that were used in this Delphi study were based on the outcomes of reviews of relevant RTW factors for comparable populations and on conversations with professionals. Often these reviews described populations with a shorter sick leave (< 2 years) or populations for which the duration of the sick leave was not mentioned or was unclear. The results of this Delphi study indicate that experts in the field recognized the majority (51 out of 58) of these factors as also being relevant in the case of long-term (> 2 years) disabled workers.

To our knowledge, there is only one other study that focused on RTW factors of long-term (> 2 years) disabled workers. In that particular study, which was also a Delphi study, Dekkers-Sánchez and colleagues investigated the perspectives of insurance physicians on RTW factors that should be included in the work ability assessment of long-term disabled workers [7]. The factors that they found to be relevant were comparable with the results of this Delphi study. The factors ‘motivation’, ‘coping’, ‘secondary gain from illness’, and ‘illness perception’ were found to be important in both studies. The factors ‘positive attitude of employee towards RTW’ did not appear in our study exactly but overlapped with other factors that were included in our study such as: ‘motivation’ and ‘job search intensity’. Other factors included in the study by Dekkers-Sánchez were more aimed at the process of RTW (e.g., providing RTW vocational rehabilitation as soon as possible or the assessment of cognition and behaviour) and were not included in the current study since our focus was not on the process of vocational rehabilitation.

#### Interventions

To inform this study, we collected VR interventions from literature reviews [22–28]. However, as discussed above, we did not find many studies that investigated which interventions were effective for workers who had been on long-term sick leave. We found four types of VR interventions that could be effective to help (long-term) disabled workers return to work: work-focused cognitive behavioural therapy (CBT) [24], motivational interviewing [23], multidisciplinary interventions [25, 26], and Individual Placement and Support (IPS) [27, 28].

From the literature it appears that IPS is effective for people with common mental disorders [27] or musculoskeletal complaints [28] to facilitate their RTW. However, the experts in our study did not recognize IPS as an effective intervention for any of the factors for the RTW of long-term disabled workers. The fact that our experts did not reach consensus on the effectiveness of IPS for any of the factors may stem from the fact that the Dutch SSI is currently only offering IPS to people with severe mental disorders. This may have led to these professionals having a limited view as to which target groups could potentially benefit from this intervention. The other three types of interventions included in the Delphi based on scientific evidence for their effectiveness (i.e., CBT, motivational interviewing, and multidisciplinary interventions) were found by the experts to be effective for some factors.

Interventions that focused on targeting psychological factors were deemed to be especially effective for many of the eight important factors for RTW. For example, “improving self-image and self-knowledge” and “increasing psychological resilience” were considered to be effective interventions for six of the factors. This shows that workers who have been out of work for a longer period are expected to benefit from interventions aimed at psychological factors.

### Strengths and limitations

Whereas previous studies focused either on which factors were important for RTW or on VR interventions that were effective (primarily in workers with a certain disease), a strength of the present study is that it aimed to connect important RTW factors to effective VR interventions. A second strength of this study was the heterogeneity of the expert panel. The three most relevant professions involved in the rehabilitation of long-term disabled workers were included in the multidisciplinary panel. The inclusion of multiple professions in this study served to establish a broader range of opinions and perspectives on the topic [31]. Another strength of this study was the high response rate on the questionnaires in the Delphi rounds. Only one of 21 participants dropped out after the first round, and only one of the remaining 20 participants missed a questionnaire round.

Unfortunately, the participation rate in the online consensus meeting was low: only half of the experts could join this meeting. This meant that half of the experts could not discuss their opinions on effective interventions with their peers. This may have led to greater difference in opinions on effective interventions between the experts that could join the meeting and the experts that could not join the meeting. However, we do not expect that this led to a bias, because no factors or interventions were selected or excluded during the meeting. Moreover, in the following questionnaire round all experts participated. Another limitation of this study might be that we did not include the long-term partially disabled workers themselves. An earlier study showed that there might be a difference in what employees, supervisors, and occupational physicians consider to be important RTW factors [32]. By not including disabled workers, we may have missed factors that are important to disabled workers for their own RTW. A final limitation is that, in the fifth round, only VR interventions for the eight most important (and influenceable) factors were presented to the experts to reach consensus. Only the VR interventions for these eight factors—which more than 70% of the experts found important for the RTW of long-term partially disabled workers—were presented. We did this to give experts the time to focus on the most important factors. However, this meant that participants could not score the effectiveness of the VR interventions for all factors for which consensus had not been reached. The results for the remaining factors would have been more accurate if they were also once more assessed by the participants in the fifth round. It could be possible that the consensus score (%) on the effectiveness of a VR intervention for a certain factor changes if it had been assessed once more.

### Implications for practice and future research

Future studies could examine which combination or cluster of factors often present together in disabled workers. A study such as this would help to develop a typology of different types of disabled workers, which could in turn contribute to developing an instrument that supports professionals.

Now multidisciplinary consensus has been reached on which RTW factors are important for the RTW of long-term disabled workers and which VR interventions are effective for this population. With this, a first step has been made in gathering evidence-based information for the development of an instrument that supports VR professionals. Such an instrument could support labour experts, caseworkers, insurance physicians, and vocational rehabilitation coaches in offering personalized VR interventions to long-term partially disabled workers. This instrument would also support professionals in assessing which RTW factors play a role in inhibiting a long-term partially disabled worker from returning to work. Likewise, it would support professionals in offering a VR intervention that targets the particular RTW factors that prohibit the worker from RTW. Finally, the instrument could improve the VR interventions received by the client by reducing practice variation in the care the client receives and improving personalized care. Additional studies, such as randomised controlled trials should verify if such an instrument is effective in facilitating the return to work of long-term disabled workers. Those studies should investigate whether such an instrument is more effective in helping long-term disabled workers return to work than usual care and whether the instrument is able to reduce practice variation among labour experts and caseworkers.

## Conclusions

This Delphi study helped to reach multidisciplinary consensus on which RTW factors are important for the RTW of long-term disabled workers, as well as which factors could be influenced using a VR intervention. In this study, consensus was also reached on which VR interventions (found in academic literature and practice) are effective for this population. The study showed that most factors which are important for the RTW of shorter-term disabled workers are often also applicable for long-term disabled workers. This Delphi also added nine new expert-based factors which were not found in previous literature but which are applicable for this group.

With this information, an instrument can be developed that supports professionals in giving evidence-based personalized care to long-term disabled workers and that could help in reducing practice variation among professionals. The effectiveness of such an instrument in daily practice should be investigated.

## Supplementary Information

**Additional file 1.**

## Data Availability

The datasets generated during and analysed during the current study are not publicly available due to privacy reasons (given the relatively small population of professionals in this field) but are available from the corresponding author upon reasonable request.

## Abbreviations

RTW: Return to work
VR: Vocational rehabilitation
SSI: Dutch Social Security Institute
CBT: Cognitive Behavioural Therapy
IPS: Individual Placement and Support
