# Supplementary material for: Return to work factors and vocational rehabilitation interventions for long-term, partially disabled workers: a modified Delphi study among vocational rehabilitation professionals
Source: BMC Public Health. 2022 May 2;22:875. doi: 10.1186/s12889-022-13295-6 (PMC9063173; doi:10.1186/s12889-022-13295-6)
Supplement: Supplementary file 1 — Additional file 1. [file 12889_2022_13295_MOESM1_ESM.pdf]

Additional file 1: supplementary material

1. Associations of factors with RTW (consensus percentage per factor per round)

#	Factor	Round 1 (%)	Round 2 (%)	Round 3 (%)	End results
1	Motivation to RTW	**100%**			**100%**
2	Disease perception	**100%**			**100%**
3	Societal participation	**100%**			**100%**
4	Importance of work	**100%**			**100%**
5	Family issues	**100%**			**100%**
6	Financial problems	**100%**			**100%**
7	Understanding of the Dutch language	**100%**			**100%**
8	Language proficiency	**100%**			**100%**
9	Social support (outside of work)	**100%**			**100%**
10	Alcohol/substance abuse	**100%**			**100%**
11	Job application skills	**95%**			**95%**
12	Coping	**95%**			**95%**
13	Job self-efficacy	**95%**			**95%**
14	Fear avoidance behaviour	**95%**			**95%**
15	Social network	**95%**			**95%**
16	Willingness to make concessions	**95%**			**95%**
17	Diplomas	**90%**			**90%**
18	Employee skills	**90%**			**90%**
19	RTW self-efficacy	**90%**			**90%**
20	Self-esteem	**90%**			**90%**
21	RTW expectations	**90%**			**90%**
22	Self-sufficiency	**90%**			**90%**
23	Quality of life	**90%**			**90%**
24	Main wage earner	**90%**			**90%**
25	Pain	**90%**			**90%**
26	Treatment	**90%**			**90%**
27	Experiences at old workplace	**86%**			**86%**
28	Unemployment	**86%**			**86%**
29	Perceived general health	**86%**			**86%**
30	Income	**86%**			**86%**
31	Work-life balance	**86%**			**86%**
32	Sense of responsibility	**86%**			**86%**
33	Willingness to learn	**86%**			**86%**
34	Disability rate	**86%**			**86%**
35	Social norms regarding RTW	**86%**			**86%**
36	Transport	**86%**			**86%**
37	Objection or appeal to decision for disability pension	**86%**			**86%**
38	Job search behaviour	**81%**			**81%**
39	Job search intensity	**81%**			**81%**

40	Volunteer work	**81%**			**81%**
41	(Re-) training	**81%**			**81%**
42	Health transition	**81%**			**81%**
43	(Informal) Care	**81%**			**81%**
44	Age	**81%**			**81%**
45	History of substance abuse	76%	**100%**		**100%**
46	Unhealthy lifestyle	76%	**90%**		**90%**
47	Work ability	76%	**85%**		**85%**
48	Previously been in contact with the law	76%	**85%**		**85%**
49	Work history	76%	**85%**		**85%**
50	Educational level	71%	**85%**		**85%**
51	Type of disabilities	71%	70%		70%
52	Driver's license	67%			67%
53	Job search assistance	57%			57%
54	Complaint	57%			57%
55	Residential area	57%			57%
56	Legal assistance	43%			43%
57	Marital status	43%			43%
58	Sex	14%			14%
59	[a] Recent life events		**100%**		**100%**
60	[a] Housing		**95%**		**95%**
61	[a] Caring for children		**90%**		**90%**
62	[a] Knowledge of the labour market		**85%**		**85%**
63	[a] Time since last working day		**80%**		**80%**
64	[a] Reintegration services already started in the past		70%	**95%**	**95%**
65	[a] Secondary gain of illness		70%	**85%**	**85%**
66	[a] Personal injury		70%	**95%**	**95%**
67	[a] Dormant employment		45%		45%

Bold: consensus reached (>80% group consensus (4) agree and (5) totally agree)

[a] Additional item added by experts in first round

2. Factors that can be targeted by using VR interventions (consensus percentage per factor per round)

#	Factor	Round 1 (%)	Round 2 (%)	Round 3 (%)	End results
1	Job application skills	100%			**100%**
2	Coping	100%			**100%**
3	Employee skills	100%			**100%**
4	RTW self-efficacy	100%			**100%**
5	Self-esteem	100%			**100%**
6	Job search behaviour	100%			**100%**
7	Motivation to RTW	95%			**95%**
8	Disease perception	95%			**95%**
9	Societal participation	95%			**95%**
10	Job self-efficacy	95%			**95%**
11	Fear avoidance behaviour	95%			**95%**
12	RTW expectations	95%			**95%**
13	Job search intensity	95%			**95%**
14	Importance of work	90%			**90%**
15	Family issues	86%			**86%**
16	History of substance abuse	86%			**86%**
17	Willingness to make concessions	86%			**86%**
18	Quality of life	86%			**86%**
19	Unhealthy lifestyle	86%			**86%**
20	Experiences in the old workplace	86%			**86%**
21	Work-life balance	86%			**86%**
22	Work ability	86%			**86%**
23	Perceived general health	81%			**81%**
24	Sense of responsibility	81%			**81%**
25	Self-sufficiency	76%	**95%**		**95%**
26	Volunteer work	76%	**90%**		**90%**
27	Previously been in contact with the law	76%	**85%**		**85%**
28	Financial problems	76%	**80%**		**80%**
29	Unemployment	76%	**85%**		**85%**
30	Social network	71%	**95%**		**95%**
31	Main wage earner	71%	75%		75%
32	Pain	67%			67%
33	(Re-) training	67%			67%
34	Willingness to learn	62%			62%
35	Job search assistance	62%			62%
36	Understanding of the Dutch language	57%			57%
37	Diplomas	57%			57%
38	Health transition	57%			57%
39	(Informal) care	57%			57%
40	Language proficiency	52%			52%

41	Disability rate	52%			52%
42	Social support (not work)	48%			48%
43	Alcohol/substance abuse	48%			48%
44	Social norms regarding RTW	48%			48%
45	Type of disabilities	48%			48%
46	Transportation	43%			43%
47	Treatment	38%			38%
48	Work history	38%			38%
49	Complaint	38%			38%
50	Income	33%			33%
51	Educational level	29%	30%		30%
52	Driver's license	29%			29%
53	Objection or appeal to decision for disability pension	29%	20%		20%
54	Age	24%	20%		20%
55	Residential area	19%			19%
56	Legal assistance	10%			10%
57	Marital status	10%			10%
58	Sex	10%			10%
59	[a] Recent life events		**90%**		**90%**
60	[a] Caring for children		**90%**		**90%**
61	[a] Knowledge of the labour market		**90%**		**90%**
62	[a] Reintegration services already started in the past		**80%**		**80%**
63	[a] Secondary gain		70%	**80%**	**80%**
64	[a] Housing		65%		65%
65	[a] Dormant employment		60%		60%
66	[a] Time since last working day		55%		55%
67	[a] Personal injury		40%		40%

Bold: consensus reached (≥80% group consensus)

[a] Additional item added by experts in first round

3. Overview of interventions

A. Informing the disabled worker about the disability benefit or re-integration process

The labour expert or caseworker informs the disabled worker about the benefit, the Dutch SSI, or the reintegration process, or points the disabled worker towards information that can be found online.

B. Professional (multidisciplinary) consultation to optimize the service offered to the disabled worker

The labour expert or caseworker can request professional consultation in order to adequately substantiate and optimize the service to the claimant. The professional consultation is multidisciplinary.

C. Assessing Vocational Needs

The labour expert or caseworker can refer the disabled worker to a service in which complex problems are translated into factors that hinder the return to work and in which insight is gained into the competencies and possibilities of the disabled worker.

D. Referral to services offered by other organizations

The labour expert or caseworker can point out to the disabled worker the services of other organizations and initiatives if there are non-work-related problems that are not addressed in the reintegration process.

E. Increasing motivation

The labour expert or caseworker sends the disabled worker to an (online) intervention or trajectory that is aimed at increasing their motivation to resume work or use motivational interviewing.

F. Improving societal participation

The labour expert or caseworker uses certain techniques in the conversation or sends the disabled worker to an (online) intervention to allow the disabled worker to participate in structured activities in an organized context to improve their societal participation.

G. Improving self-image and self-knowledge

The labour expert or caseworker sends the disabled worker to an (online) intervention that is aimed at improving the worker's self-image and self-knowledge or works on this with the disabled worker directly. The intervention can be aimed at discovering one's strengths and weaknesses, improving one's self-image, and processing barriers that stem from a low self-image.

H. Increasing psychological resilience

The labour expert or caseworker sends the disabled worker to an (online) intervention or process that is aimed at improving psychological resilience or works on this aspect with the disabled worker directly.

I. Improving vitality and physical resilience

The labour expert or caseworker sends the disabled worker to an (online) intervention or trajectory that is aimed at improving vitality and physical resilience or works on this aspect with the disabled worker directly.

J. Strengthening employee skills

The labour expert or caseworker sends the disabled worker to an (online) intervention or process that is aimed at strengthening employee skills or works on this in meetings with the disabled worker.

K. Identifying what the disabled worker wants to do in terms of work

The labour expert or caseworker sends the disabled worker to an (online) intervention or process that is aimed at identifying what the disabled worker wants in terms of work or helps the disabled worker identify this themselves.

L. Identifying what the disabled worker can do in terms of work

The labour expert or caseworker sends the disabled worker to an (online) intervention or process that is aimed at making an inventory of what the disabled worker can do in terms of work or helps the disabled worker to look for chances of finding their preferred profession in the regional labour market.

M. Helping to search for vacancies

The labour expert or caseworker sends the disabled worker to an (online) intervention or trajectory that is aimed at helping to search for vacancies or asks the disabled worker to work on this themselves.

N. Improving skills and helping with applying for a job

The labour expert or caseworker sends the disabled worker to an (online) intervention or trajectory that is aimed at helping with applying for a job or helps the disabled worker with this directly.

O. Mediating

The labour expert or caseworker mediates in finding a job or sends the disabled worker to an (online) intervention or process that mediates in finding a job.

P. Workplace adjustments or support

The labour expert or caseworker helps the worker with adjusting the job content, the workplace or working conditions or sends the disabled worker to an (online) intervention for this.

Q. Training

The labour expert or caseworker can refer the disabled worker to training in order to acquire work and/or labour market relevant knowledge and/or skills.

R. Increasing work experience

The labour expert or caseworker can refer the disabled worker to a traineeship where a disabled worker can follow a (vocational) training course. A reintegration company assists the disabled worker.

S. Providing facilities

The labour expert or caseworker can request facilities, such as a job coach or a sign language interpreter, so that the disabled worker can resume work.

T. Cognitive behavioural therapy

Cognitive behavioural therapy can be used to adjust unhelpful irrational thoughts, ie. thoughts that lead to dysfunctional behaviour, such as avoidance or aggression. An example of cognitive behavioural therapy is Acceptance and Commitment Therapy, which aims to increase functional abilities and quality of life instead of reducing complaints.

U. Multidisciplinary interventions

Multidisciplinary interventions are interventions that look at a person from multiple specializations. Each specialization assesses which possibilities and obstacles someone has in returning to work. Based on these assessments, a personalized reintegration plan is drawn up to get started.

V. Individual placement and support (IPS)

This intervention is aimed at placing people who are motivated to work in paid employment as soon as possible, whereby the person is (intensively) guided at the workplace by a job coach. This is the so-called first-place-then-train principle.
